# Extending voluntary health insurance to the informal sector: experiences and expectations of the informal sector in Kenya

**DOI:** 10.12688/wellcomeopenres.12656.1

**Published:** 2017-09-28

**Authors:** Edwine W. Barasa, Njeri Mwaura, Khama Rogo, Ledia Andrawes

**Affiliations:** 1 Health Economics Research Unit, KEMRI Wellcome Trust Research Programme, Nairobi, Kenya; 2 Health in Africa Initiative, The World Bank Group, Nairobi, Kenya; 3 Institute of Global Prosperity, University College London, London, UK

**Keywords:** Universal Health Coverage, social health insurance, informal sector

## Abstract

**Background**: Kenya has made a policy decision to use contributory health insurance as one of its key pre-payment health financing mechanisms. The National Hospital Insurance Fund (NHIF) is the main health insurer in Kenya. While the NHIF has hitherto focused its efforts on providing health insurance coverage to individuals in the formal sector, it has recently broadened its focus to include individuals in the informal sector. This paper provides an analysis of the perceptions, and experiences of informal sector individuals in Kenya with regard to enrolment with the NHIF.

**Methods**: We collected data through key informant interviews (39) in two purposefully selected counties. Study participants were drawn from healthcare facilities contracted by the NHIF, and current, former, and prospective informal sector members. We analyzed data using a grounded approach.

**Results**: Participants felt that the NHIF provided inadequate information about the registration and membership processes as well as benefit entitlements. There was variable and inconsistent communication by the NHIF. There was also variance between the official benefit package and the actual benefits received by members. The NHIF registration requirements and processes presented an administrative barrier to obtaining membership. The NHIF premium level and contribution mechanism presents a financial barrier to current and prospective members. Healthcare providers discriminated against NHIF members compared to cash-payers or private insurance holders.

**Conclusions**: The NHIF could improve enrolment and retention of informal sector individuals by; 1) using communication strategies that are effective at reaching the informal sector, 2) improving the affordability of the premium rates, 3) simplifying the enrolment requirements and process, and 4) strengthening accountability mechanisms between itself and healthcare facilities to ensure that enrolled members receive the benefits that they are entitled to, and that client experience at healthcare facilities are satisfactory.

## Introduction

Low and Middle Income Countries (LMICs) are increasingly prioritizing the attainment of Universal Health Coverage (UHC) ( Sachs, 2012). The goal of UHC is to ensure that everyone has access to healthcare services that they need, of good quality, without the risk of financial ruin or impoverishment ( WHO, 2010). This commitment has culminated in the inclusion of UHC in the Sustainable Development Goals (SDGs), which were adopted by world leaders in 2015 to articulate global development priorities until 2030 ( United Nations, 2015). To achieve UHC, countries must expand the range of services they provide to their citizens, expand population coverage with a pre-payment mechanism, and reduce the proportion of direct costs that citizens pay to access healthcare services ( Chan, 2016).

Mandatory health insurance is gaining increasing attention as a health financing mechanism in LMIC countries reforming their health systems for UHC ( Lagomarsino *et al.*, 2012). However, international evidence has shown that it is problematic to achieve high coverage among the informal sector using a voluntary, contributory mechanism for several reasons ( Lagomarsino *et al.*, 2012; McIntyre *et al.*, 2013). One, a significant proportion of informal workers are less well-off, compared to formal sector workers and therefore have a lower ability-to-pay for health insurance ( Alkenbrack *et al.*, 2013; Oxfam, 2013). Two, given that the informal sector is not organized in sizeable groups, it is administratively difficult to recruit, register and collect regular contributions in a cost effective way. Membership and premium payment is therefore often voluntary leading to low uptake, poor retention and adverse selection ( Jowett, 2015; Lagomarsino *et al.*, 2012). Three, informal sector worker incomes are often unpredictable, ( Lagomarsino *et al.*, 2012), which makes it difficult to collect premiums regularly and increases attrition rates among this population. Voluntary insurance contributions therefore present a fairly small percentage of overall health revenues, even in countries that continue to attempt to collect them ( Jowett, 2015; McIntyre *et al.*, 2017). Despite these challenges, an increasing number of Sub-Saharan African countries have either established, or are in the process of establishing a contributory public health insurance scheme. For example, Ghana, Kenya, Nigeria, Rwanda and Tanzania have contributory public health insurance schemes, while South Africa, Swaziland, Lesotho, Sierra Leone, Liberia, Zambia, Uganda, Bukina Faso and Zimbabwe are considering establishing one ( Josephson, 2017; Lagomarsino *et al.*, 2012; Tetteh, 2012).

Kenya has made a commitment to achieve UHC by 2030 ( Ministry of Medical Services, 2012). The country has a mixed health financing system that is financed through public, private, and donor resources. According to the latest national health accounts, donors, public sources, and private sources accounted for 25.6%, 33.5%, and 39.8% respectively, of the country’s total health expenditure (THE) in 2013 ( Ministry of Health, 2015). Public sources of revenue are collected by a mix of direct and indirect taxes, and premium contributions to the National Hospital Insurance Fund (NHIF). The NHIF is a government corporation that was established in 1966 to provide mandatory health insurance to formal sector employees ( IFC, 2011), and its mandate later expanded to cover informal sector workers in 1998 ( IFC, 2011). Health insurance coverage in Kenya remains low at 19.59 ( Kazungu & Barasa, 2017); 88.4% of those with health insurance are covered by the NHIF, while 11.6% are covered by private insurers ( Ministry of Health, 2014). The low health insurance coverage is perhaps not surprising given that Kenya, like most LMIC countries has a disproportionately high informal sector population. According to a world bank report, while on average 728,000 new jobs were created in Kenya in 2016, 632,000 (87%) were in the informal sector ( World Bank Group, 2016). Out of pocket payments (OOP) formed the greatest proportion of private health financing sources; OOP spending as a proportion of THE was 29% in 2013 ( Ministry of Health, 2015). As a result of this high reliance of OOP, 4.52% of the Kenyan population incur catastrophic health expenditures, while 453,470 individuals are pushed into poverty annually because of healthcare payments ( Barasa *et al.*, 2017).

The Kenyan government has made a decision to use the NHIF as one of the key strategies for scaling up population coverage with a prepayment health financing mechanism ( Ministry of Medical Services, 2012; Munge *et al.*, 2017). This means that the government of Kenya, and the NHIF have to confront the informality problem and develop strategies to expand coverage among the informal sector. Membership to the NHIF is mandatory for formal sector workers, who pay an income rated monthly contribution through statutory deductions, while it is voluntary for informal sector workers who pay a flat rate contribution directly to the NHIF. In an effort to expand health insurance coverage, the NHIF has in the recent past implemented a number of reforms. These include the introduction of an outpatient benefit package. Previously, the NHIF offered an inpatient only benefit package to the public. Expanding coverage to include outpatient services is thought to be a strategy to make the NHIF more attractive to the public and hence drive enrolment. To enable this benefit package expansion, the NHIF revised its premium contribution rates upwards ( Table 1) ( GIZ, 2016). Prior to this revision, the NHIF premiums were last revised in 1988. The monthly contributions for the lowest paid formal employee increased by 167 percent, while rates for the highest earners increased by 431 percent. Contribution rates for the informal sector increased by 213% ( Gok, 2015).

**Table 1.** Revisions of NHIF contribution rate.

OLD RATES (KSH)		NEW RATES (KSH)		% INCREASE
INCOME GROUP	PREMIUM	INCOME GROUP	PREMIUM	
1,000-1,499 (US$ 13-20)	30 (US$0.4)	Less than 5,999 (Less than (US$80)	150 (US$2)	400
1,500-1,999 (US$ 20-27)	40 (US$0.5)			275
2,000-2,999 (US$ 27-40)	60 (US$0.8)			150
3,000-3,999 (US$ 40-53)	80 (US$1)			88
4,000-4,999 (US$ 53-66)	100 (US$1.3)			50
5,000-5,999 (US$ 66-80)	120 (US$1.6)			25
6,000-6,999 (US$ 80-93)	140 (US$2.1)	6,000-7,999 (US$80-106)	300 (US$4)	114
7,000-7,999 (US$ 93-106)	160 (US$2.1)			88
8,000-8,999 (US$ 106-120)	180 (US$2.4)	8,000-7,999 (US$106-159)	400 (US$5.3)	122
9,000-9,999 (US$ 120-133)	200 (US$2.6)			100
10,000-10,999 (US$ 133-146)	220 (US$2.9)			82
11,000-11,999 (US$146-159)	240 (US$3.2)			67
12,000-12,999 (US$ 159-172)	260 (US$3.5)	12,000-14,999 (US$159-199)	500 (US$6.7)	92
13,000-13,999 (US$172-186)	280 (US$3.7)			79
14,000-14,999 (US$186-199)	300 (US$4)			67
15,000 and above (US$199 and above)	320 (US$4.3)	15,000-19,999 (US$199-266)	600 (US$8)	88
		20,000-24,999 (US$266-332)	750 (US$10)	134
		25,000-29,999 (US$332-398)	850 (US$11.3)	166
		30,000-49,999 (US$398-664)	1,000 (US$13)	213
		50,000-99,999 (US$664-1328)	1,500 (US$20)	369
		Over 100,000 (Over 1328)	2,000 (US$27)	525
**Voluntary/Self Employed**	**160 (US$2.1)**	**Voluntary/Self Employed**	**500 (US$6.7)**	**213**

This paper reports findings of a study that examined the experiences and perceptions of informal sector individuals with regard to membership with the NHIF. Findings from this study are potentially useful in informing country level strategies for scaling up NHIF membership in the population, and in similar LMIC contexts that are either planning to or employing voluntary contributory health insurance as a mechanism for providing population coverage with a prepayment health financing mechanism.

## Methods

We used a qualitative cross-sectional study design, and collected data through in-depth interviews. The approach was exploratory, where we aimed to discover the perceptions, and experiences that affect an informal sector person’s willingness to enroll and to remain an NHIF member. The NHIF considers individuals who are self-employed in small scale business, and those who have casual employment arrangements that are typically not on payrolls or taxed, as belonging to the informal sector. The distinguishing feature in their relationship with the NHIF is that their premium contributions are not deducted at source by their employers and remitted to the NHIF. Rather, the individuals have to voluntarily pay premiums to the NHIF by themselves. We used this definition for the informal sector in our study.

We collected data in two purposely selected counties. We considered the poverty levels and degree of urbanization in the selection of the two counties. One of the counties selected had a lower poverty incidence and was predominately urban, while the other had a higher poverty index and predominantly rural. We carried out a total of 39 in-depth interviews. Table 2 outlines the number of participants selected for interviews.

**Table 2.** Number of study participants.

In-depth interviews		
	Kiambu County	Makueni County
NHIF contracted healthcare facilities	2	2
Current informal sector members of the NHIF	6	4
Former informal sector members of the NHIF	6	3
Informal sector individuals that have never enrolled with the NHIF	9	6
**Total number of interviews**	**39**	

At the county level, we selected study participants from healthcare facilities contracted by the NHIF, and individuals from the informal sector. For the in-depth interviews with informal sector individuals, we drew participants from individuals who were a) current NHIF members, b) former NHIF members, and c) never been NHIF members. We purposefully selected current and former NHIF informal sector members from the NHIF member’s database. We then called people on the list, asked some preliminary screening questions (to ensure a spread of age, gender and informal work) and introduced the study over the phone before inviting them for a face to face interview. We selected informal sector individuals who had never previously enrolled with the NHIF by first engaging two dedicated community mobilisers in each county. The community mobilisers identified individuals from various walks of life through visits to market places and community institutions, qualifying potential participants through the preliminary screening questions, briefing them and then scheduling them in for the interviews. The interviews lasted between 45–60 minutes each. We audio recorded the interviews and supplemented with note taking.

### Data analysis

We imported manually transcribed data into Microsoft Excel software for coding and analysis. Data were analyzed using a grounded approach. First, we read through the transcripts to familiarize with the data. Second we conducted open coding where key phrases or ideas were identified and labelled as the key thematic categories. Third, we conducted axial coding where we associated open codes and created sub categories of emerging themes and concepts. Fourth, we charted the coded data and the emergent thematic categories. Interpretation of the data entailed identifying key concepts and explaining relationships between these key concepts.

### Ethical statement

The authors received ethical approval from the Africa Medical Research Foundation (AMREF) ethics and scientific review committee (approval number ESRC P168/2015). Written informed consent to collect the data, and to publish the findings based on the data provided by the participants was obtained from all study participants.

## Results

### Inadequate and inconsistent communication by the NHIF

Participants felt that the NHIF did not effectively and consistently communicate and provide information to its members and the general public (including current and prospective members). This included information on registration requirements and processes, service entitlement, network of healthcare providers that provided services for its members, premium payments and penalties on default. When individuals actively sought information from the NHIF, the information was inconsistent across different NHIF branches, and NHIF officials. Participants felt that this inadequate information was an entry barrier to prospective members, and a source of frustration for existing members.

*“What made me not join is not having someone to tell me about it. Like now to be a boda-boda [motorbike taxi] rider you need someone who knows how to ride one and teach you how to ride. The same thing with NHIF, I need someone who knows about it to tell me about it” – Potential member*

*“It is important to know especially as a contributor what NHIF is offering. They should let people know these things; where to pay, what they are covered for and if their family is sick, where do they get assistance?” – Former member*

### The NHIF registration requirements and processes presented a barrier to membership

The NHIF registration process was long, complicated and required documents that potential members sometimes did not have. Participants felt that the NHIF registration requirements are too rigid and difficult for some to meet, and they therefore become a deterrent to uptake of membership. For example, it is not possible for some to provide birth certificates for their children. This may be because the child does not have a birth certificate. Other difficulties include those who need to add orphaned children they have adopted.

*“I once tried applying for NHIF membership and was asked for birth certificates, my ID card and that of my wife, passport photos and Kshs 480. I never went back there” – Potential member*

*“I tried to get my three grandchildren to join since their mother passed away and I had the birth certificate and so they asked me to produce their birth certificates, and documents confirming that and then get a letter from the chief, children’s office, and the police station stating that the children are in my custody and I felt it was a long process so I stopped so I don’t know why I am still paying” – Current member*

*“My husband went to the branch and he was given an application form but it had very difficult questions, he looked at it and threw it away and asked me to forget about it (NHIF)”*

*– Potential member*

### The NHIF premium level and contribution mechanism presents a financial barrier to current and prospective members

Participants felt that the NHIF premium payment mechanism imposed a significant barrier to current and prospective members. First, at the time of data collection, the NHIF had just announced an upward revision of premium contribution rates (table x). Under the new premium contribution structure, the monthly premium contribution rates for members in the informal sector had been revised upwards from KES 160 (USD 1.6) to KES 500 (USD 5). This represented a 213% increase in monthly premium contribution rate. This new rate was thought to be unaffordable. The new premium rate was also thought to be unfair and inequitable since it was a flat rate rather income rated. This rate was thought to be disproportionate to the income levels of the majority of the informal sector members. While the NHIF had subsidy programmes for the poor and vulnerable (including poor, orphans and vulnerable children, the disabled, and the elderly), it was reported that most informal sector individuals and households did not meet the criteria to be included in these programmes.

*“People already have a problem paying 160 per month I don’t see how they will be able to pay 500 per month” – Potential member*

*“Let it be 160 times 2 which is equal to 320Ksh, because it will be affordable” – Former member*

*“[The contributions] should not go that high level, that maybe some people who are earning very little amounts of money each and every month won’t be able to continue paying or join” – current member*

Second, participants felt that regular, monthly payments were challenging for those with fluctuating and irregular incomes. Many would prefer more flexible payment terms. For example, those who had more seasonal incomes would like a system that allowed them to make payments when they can afford. For others, whose income was daily, or weekly based, a shorter-term payment option such as day-to-day was preferred.

*“For me I can pay per week because I get money daily” – Potential member*

*“My income is inconsistent; I feel that [NHIF] is good for employed people since its deducted from their salaries” – former member*

Third, the default penalty is too high and was a significant barrier for re-entry. Many current and former members, felt that the main reason they may default was due to financial hardship; the penalty was seen as a major barrier for continuing membership if they were to default.

*“My worry is always about penalties that is the first thing because at times you might even pay 4 years then go through a rough path and fail to pay for 2 to 3 months then you realize the penalty is Kshs 6,000. It is too difficult for me to get it. The penalty makes people fear for such commitments” – Current member*

*“It is a challenge paying that penalty because in the first place you default because of lack of money” – Current member*

*“When you fail to pay…[NHIF] should understand there was an issue. You have been contributing and they stopped abruptly they should ask what happened not slap you with a penalty” – Former member*

Further, current and former members reported that they found it very difficult to remember when to pay, resulting in accidental missed payments and consequently, penalty and the suspension of their membership. While some took proactive measures to remember, such as calendar reminders, most stated that they would find an SMS reminder system highly valuable for maintaining their payments.

*They should remind us people in the informal sector. They tell us, remember you have to pay your NHIF due by this time. Your account is expiring” – Current member*

*“They should automate their systems for mobile updates, reminders, as well as package notifications” – current member*

The NHIF had made an attempt to improve the convenience of premium payments by introducing a mobile money payment system. However, it was reported that this payment system was not always reliable. Often, NHIF members would pay their premiums through the mobile money payment system, but the payment was not reconciled with the members account. Such members would be considered payment defaulters and would be denied access to services.

*“At times you pay and then when you go to check you find that the money does not reflect” – Current member*

*“My brother was admitted to hospital, he was paying via M-PESA but still it had not gotten into the system. His wife was told by the call center to go to the headquarters to complain about that money. In the computer, it was showing that she had not paid, but she had those messages she got after paying. She had to queue for long. She was told to write a letter and say what she used to pay via the phone” – Current member*

### Healthcare providers discriminated against NHIF members

Participants reported discrimination by healthcare providers. This discrimination took different forms. First, it was reported that healthcare providers preferred providing services to either cash paying clients or those covered by private insurers. Healthcare providers felt that the reporting requirements of the NHIF were burdensome, and also complained about delays by the NHIF in processing claims and making payments to providers.

*“Discrimination of clients does happen in some facilities if you are an NHIF member…The way NHIF treats the facility is the cause. Linda Jamii [private insurer] and Britam [private insurer] have paperless claims process and they always pay at the end of the month. Most of the time NHIF reimburses late. Other times they don’t pay the full amount. Like when you process a claim worth one million they only pay two hundred thousand. I think there is someone there who is just slow.”– NHIF contracted facility*

*“When they see you using the card, the patient is not given good care. I was abandoned there in [a public hospital] from morning to evening because we had brought an NHIF card. So when we gave them the card they neglected us” – Current member*

*“I visited somebody in [the] district hospital, she had given birth and because she was catered for by the [NHIF] card, she was sent home immediately after giving birth and those who were paying cash would stay two or three days” – Potential member*

Second, among NHIF members, healthcare providers preferred providing services to members of the civil servant’s scheme, over members of the general scheme. This was because the general scheme, to which informal sector members belonged, paid lower inpatient and outpatient rates to healthcare facilities compared to the civil servant’s scheme. Further, the NHIF had a generous benefit package for civil servants and a narrow benefit package for everyone else. For example, civil servants had a benefit package that included air rescue, and treatment abroad. Civil servants could also seek services in private facilities without balance billing; the NHIF met all the costs of care in these facilities. The rest of NHIF members did not have such generous benefits and had to make co-payments in private facilities.

*“Civil servants pay the same as everyone else – Ksh 320 max. But they get way more services. Why are they taking our money to do that? Why does NHIF favor public servants?” – Current member*

*“Civil servants get special attention like doctor’s visits and clean sheets. The NHIF is really only for this special class. These rich guys [civil servants] get access to a doctor two times a day unlike the normal ones [members of the general scheme) who access the doctor once” – Former member*

### Variance between the official benefit package and the actual benefits received by members

Participants reported that while the NHIF benefit package was on paper comprehensive and attractive to them, the range of benefits they received in practice was limited and unattractive. For example, while the NHIF official guidelines on the benefit package stated that members were to receive comprehensive inpatient and outpatient care, certain services were often not available in the healthcare providers that NHIF had empaneled to provide services to its members. This included medicines, laboratory, and radiological tests. NHIF members were hence forced to seek these services in non-NHIF empaneled facilities and pay for them out of pocket. Further, while the NHIF inpatient benefit package was meant to be comprehensive, healthcare providers felt that the daily rebate paid by the NHIF was inadequate and hence balance billed NHIF members.

*“So when [the facility] heard about the [NHIF] card they tried to increase the charges on what we had to pay cash. So they tried to get as much money from us that was not covered by the card” – Current member*

*“We hear in the media that the medication in public hospitals is free, but when you go there, the first thing you will be requested to do us to pay Kshs 100 even before you see a doctor” – Potential member*

## Discussion

This study presents the experiences and perceptions of individuals in the informal sector in Kenya, about the NHIF. The analysis shows that the enrolment and retention of informal sector individuals into the NHIF is influenced by both purchaser (NHIF) factors, and provider (healthcare facilities) factors. One of the purchaser factors is the inadequacy of communication and information sharing between the NHIF and the public. The public is not adequately aware about how to join the NHIF, how to access services as an NHIF member, and what their entitlements are as NHIF members. This finding is interesting considered against a background of sustained social marketing and advertising campaigns by the NHIF. It is likely that while the NHIF invests in marketing initiatives, the avenues chosen, such as bill board advertisements, road shows, TV and radio, are not reaching informal sector worker in rural areas, who likely do not have access to these channels of communication. Inadequate information has been shown to present a barrier to insurance enrolment not just in Kenya ( Mathauer *et al.*, 2008) but in other similar contexts. For example, a study of determinants of enrolment to a public voluntary health insurance scheme in South Africa reported that 24% of participants identified lack of information about the scheme as an important barrier to enrolment ( Govender *et al.*, 2013). Another purchaser factor is the administrative obstacles associated with procedures and requirements for enrolling the NHIF. These processes are long, complex and inconveniencing to individuals with a desire to enroll. For example, the requirement for birth certificates and national identity cards is quite challenging in settings where the majority of the population are poor, uneducated, and leave in rural areas where birth notification and civil registration rates are low. In Kenya, the birth registration rate is only 57% in rural areas, and 81% in urban areas ( UNICEF, 2017). Administrative obstacles to enrolment have been reported in South Africa ( Govender *et al.*, 2013) and Ghana ( Jehu-Appiah *et al.*, 2012). Lastly, participants identified the cost of premium payments to have NHIF insurance cover, the penalties on default, and the inflexibility of payment plans as key barriers to individuals in the informal sector. This finding is similar to those in other settings. For example, in Ghana, informal sector individuals living in rural areas found it difficult to enroll to the National Health Insurance service because they had low ability to pay ( Agyepong *et al.*, 2016).

Apart from the purchaser specific factors, provider factors were also identified. Getting individuals to register and belong to a health insurance scheme is only but one aspect, ensuring that individuals with health insurance cards have access to care and that their care seeking experience is appropriate is equally important. When the services that individuals belonging to an insurance scheme do not match with their formal entitlements, and when they are treated disrespectfully and/or discriminated against by healthcare providers, it leads to attrition. It also generates a negative perception about joining the health insurance scheme, which in turn deters new member registrations ( Agyepong *et al.*, 2016; Alhassan *et al.*, 2016; Jehu-Appiah *et al.*, 2012). As a healthcare purchaser, it is imperative that the NHIF strengthens the accountability mechanism with healthcare providers. Weakness in the agency relationship between the NHIF and healthcare providers have also been identified by others ( Munge *et al.*, 2017).

Drawing from this, we make the following recommendations for policy:

1. The NHIF should review its communication and awareness creation strategies to identify mechanisms that are effective at reaching the informal sector. For example, they could borrow strategies from local immunization programmes which combine local media messages with community outreach by community health workers.
2. The NHIF, and more broadly the government of Kenya, should consider the affordability of its contribution premiums to the informal sector, as well as the flexibility of payments. One option would be to consider introducing partial subsidies that are tax funded to reduce the financial burden imposed by high premium contribution rates.
3. The NHIF should review its registration requirements and procedures so as to reduce the complexity of registration and the burden of registration requirements. In a context where a significant proportion of the informal sector do not have national identity cards and birth certificates, the NHIF could consider using alternative forms of identification such as referrals from local leaders, and local community based organizations.
4. The NHIF should proactively and regularly obtain information on client experiences at healthcare facilities and use this information to take action that will dis-incentivize health facilities from discriminating against NHIF members. This could include identifying and resolving the factors that make health facilities discriminate against NHIF members. These include resolving delays and unpredictability of claims processing and payments to healthcare facilities. Incorporating client feedback into a quality assessment that is linked to NHIF provider payment rates could also disincentive healthcare facilities from discriminating against NHIF members.
5. The NHIF should align the formal benefit package that its members are entitled to and what its members actually receive when they visit healthcare facilities. One of way of doing this is by making explicit the benefit package and implementing a system for monitoring healthcare facilities to ensure that they deliver the formal benefit package. The NHIF could, for example, adopt the model used by some private insurers, where “care managers” are assigned to contracted hospitals with the responsibility of monitoring care provision to its members.

## Data Availability

The data referenced by this article are under copyright with the following copyright statement: Copyright: © 2017 Barasa EW et al.

Interview transcripts that contain the study data are available on the OSF data repository under the project title ‘NHIF informal sector perceptions study’ ( http://dx.doi.org/10.17605/OSF.IO/82BJU) ( Barasa, 2017). The transcripts have been redacted, and de-identified to preserve the anonymity of the study participants.
