# Supplementary material for: Extending voluntary health insurance to the informal sector: experiences and expectations of the informal sector in Kenya
Source: Wellcome Open Res. 2017 Sep 28;2:94. [Version 1] doi: 10.12688/wellcomeopenres.12656.1 (PMC5698913; doi:10.12688/wellcomeopenres.12656.1)
Supplement: Peer review history

# Peer review: Referee response for version 1

**DOI:** 10.21956/wellcomeopenres.13703.r27318

**Published:** 2017-11-30

**Authors:** Gilbert Abotisem Abiiro

**Affiliations:** 1 Department of Planning and Management, Faculty of Planning and Land Management, University for Development Studies, Wa, Ghana

This is a well-constructed and well-written paper. It is methodologically rigorous and scientifically sound. My only concern is that the challenge of using contributory health insurance to cover the informal sector has been topical in the global universal health coverage debates. After reading about the Kenyan experience in this paper, I expected the authors not only to make recommendations for improving health insurance coverage within the informal sector of Kenya alone but to also advise the global health policy and research community about the prospects of contributory health insurance as a strategy for universal health coverage of the informal sector. This comment is rooted in the fact that all the recommendations presented in this paper are targeting the Kenya Health Insurance Authorities and the Kenyan Government.

I have read this submission. I believe that I have an appropriate level of expertise to confirm that it is of an acceptable scientific standard.

# Peer review: Referee response for version 1

**DOI:** 10.21956/wellcomeopenres.13703.r26627

**Published:** 2017-11-21

**Authors:** Michelle Remme

**Affiliations:** 1 Department of Global Health and Development, London School of Hygiene & Tropical Medicine, London, UK

This is a clear and well-written paper that provides important insights into the challenges of extending voluntary health insurance to the informal sector in Kenya. The background and justification for the study are compelling, and the implications of the findings for Kenya are well articulated.

 That being said, certain sections would require further elaboration or clarification:  

- The study purposively selects two counties with different poverty levels and degrees of urbanization, but the analysis does not exploit this or indicate whether there were any significant differences in findings.
- The findings report inequities in the official benefits and premium levels for civil servants and other formal sector or informal sector members. This point has been merged under the sub-section on health care providers’ reported discrimination against NHIF members, but it appears quite distinct. Indeed, if informal sector payers are paying similar premia, but are officially entitled to a more limited benefit package, this seems to be a critical design issue that will impact on their willingness to join the scheme.
- The discussion section is very specific to Kenya, including context-specific recommendations. While it clearly makes sense to draw out the implications for the NHIF in Kenya, it would be useful to broaden the discussion somewhat and consider policy implications for other settings. The introduction underscores the relevance of the study, given efforts in several countries to extend universal health coverage through national schemes that include voluntary insurance for the informal sector, yet the discussion does not place the findings back in this context.
- In the methods section, the selection of counties could be explained in more detail. Also, it would be useful to provide a summary of the characteristics of the respondents, besides whether they are current, former or potential NHIF members (age, marital status, occupation, etc).  
- The authors quote a health care provider in the results section, but this category of respondents is not mentioned in the methods section.

I have read this submission. I believe that I have an appropriate level of expertise to confirm that it is of an acceptable scientific standard.

# Peer review: Referee response for version 1

**DOI:** 10.21956/wellcomeopenres.13703.r27317

**Published:** 2017-11-08

**Authors:** Timothy Abuya

**Affiliations:** 1 Reproductive Health Program, Population Council, Nairobi, Kenya

General comments

 The paper is well written and reflects the contextual challenges of access of service via NHIF among the voluntary general scheme.

 The data is well presented and provides deeper insights on the key barriers to involving the informal sector users.

 However, there are a few issues that the authors could consider:

1. Although the authors used the informal sector derived from NHIF, I feel inclusion of the fact that they are not taxed may be misleading. Perhaps qualifying it further would be useful
2. The authors also note that they used grounded approach in the analysis, I presume they must have designed it first deductively be identifying areas of examination a priory. There may be need to clarify that they both used inductive and deductive approaches in the methods section. Use of grounded approaches as stated may suggest potential design that is theory driven. I think they used thematic content analysis that is was both deductive and inductive driven
3. In the result section although well described and presented there are opportunities for linking the results that speak to each other. For example, inadequate and inconsistent communication by NHIF affects understanding of the NHIF registration requirements limiting desire and access to NHIF inclusion.
4. In terms of the discrimination- can the authors clarify that the preference of provision of services to civil servants was reported in public health sector or private sector? This is important for readers to make judgment of the results and its implications for making changes.
5. The results presented are against a backdrop of challenging context where public sector providers have had several industrial strikes and changes in managing health care in devolve structures, a mention of this background would be useful to the readers as they may explain some of the result around variance of services on paper versus those that are provided.
6. The authors make a recommendation on subsides for that are tax funded, can they also provide a rider that targeting mechanism can be tricky and may require investment to ensure subside reach the targeted communities.

I have read this submission. I believe that I have an appropriate level of expertise to confirm that it is of an acceptable scientific standard.
